# From mat to sand: a comparative injury surveillance study of Olympic and Beach Wrestling at the 2025 World championships

**DOI:** 10.1186/s40621-026-00664-7

**Published:** 2026-02-27

**Authors:** Szabolcs Molnár, Dorsaf Metahni, Saam Falahati, Francisco Lee, Radivoj Filipov, Babak Shadgan

**Affiliations:** 1 Medical, Prevention and Anti-Doping Commission, United World Wrestling, Vevey, Switzerland; 2 Orthopedic-Trauma Department, Central Hospital of Northern Pest - Military Hospital, Budapest, Hungary; 3 Sports Medicine and Sciences Center, Sousse, Tunisia; 4 Al Qadsiah Sports Club, Al Khobar, Saudi Arabia; 5 Vivit GT Rehabilitation Clinic, Guatemala City, Guatemala; 6 Department for Disease Control and Prevention, Institute of Public Health, Zrenjanin, Serbia; 7 Department of Orthopedics, University of British Columbia, 49 Deerwood Place, Port Moody, Vancouver, BC V3H4X7 Canada

**Keywords:** Beach Wrestling, Olympic Wrestling, Injury epidemiology, Combat sports, Athlete safety, Injury severity, Sports trauma, Injury surveillance, Prevention

## Abstract

**Background:**

Wrestling is associated with a substantial risk of injury; however, comparative injury epidemiology between Olympic Wrestling (OW) and Beach Wrestling (BW) remains limited, constraining evidence-informed injury-prevention strategies across formats.

**Methods:**

A prospective comparative epidemiological study was conducted during the 2025 Senior Olympic Wrestling World Championships and the 2025 Beach Wrestling World Series. Only competition-related bout injuries requiring on-site medical assessment or treatment were recorded. Injuries were documented using the United World Wrestling (UWW) standardized injury report form and expressed as incidence rates per 1,000 athlete-exposures (AEs). Injuries were classified by severity, anatomical region, and wrestling format. Between-group comparisons were performed using chi-square tests and independent-samples t-tests (*p* < 0.05).

**Results:**

A total of 53 competition-related injuries were recorded: 29 in BW (20.0 injuries per 1,000 AEs) and 24 in OW (13.3 injuries per 1,000 AEs). Although BW demonstrated a higher overall injury incidence, all BW injuries were classified as mild or moderate, with no severe or catastrophic cases. In contrast, OW demonstrated a significantly higher proportion of moderate-to-severe injuries compared with BW (75% vs. 14%; χ² = 14.73, *p* < 0.001). The head and face were the most commonly affected regions in both formats (38% in BW; 42% in OW). Secondary injury patterns differed, with OW injuries more frequently involving the knee (25%) and shoulder (17%), whereas BW injuries were more evenly distributed across the upper extremities (34.6%). No catastrophic injuries occurred in either format.

**Conclusions:**

Beach Wrestling demonstrated a more favourable injury-severity profile despite a higher overall injury incidence, whereas Olympic Wrestling was associated with a greater proportion of clinically significant injuries. These findings highlight important format-specific differences in injury patterns and support the need for targeted injury-prevention strategies and continued evaluation of competition rules and environments.

## Background

Wrestling is one of the oldest organized combat sports and remains a core discipline in international competition, including the Olympic Games. Its dynamic and high-intensity nature, characterized by rapid changes in direction, high-force throws, joint locks, and sustained grappling, places considerable biomechanical demands on athletes and contributes to a well-documented risk of musculoskeletal injury [1–3]. Previous epidemiological studies have identified injuries involving the knee, shoulder, elbow, and head or face as the most common in Olympic-style wrestling (OW), with outcomes ranging from minor contusions to ligament tears, fractures, and concussions [4–6]. Understanding injury patterns in wrestling is therefore essential for advancing athlete safety, informing evidence-based prevention strategies, and improving sport-specific medical care [7, 8].

Beach Wrestling (BW) is a more recent competitive format, governed by United World Wrestling (UWW), developed to enhance global accessibility and spectator appeal. Unlike traditional OW, which is performed on a sprung mat and features two three-minute periods, BW takes place on natural sand, follows a single three-minute bout, and employs simplified scoring that rewards takedowns, pushouts, and controlled throws [9]. These structural differences, including surface compliance, shorter match duration, restricted contact rules, and absence of prolonged par terre wrestling, create a competition environment that may alter the biomechanical loading and injury exposure profile. While OW has been extensively studied, with robust injury surveillance at the Olympic Games and world championship competitions [2, 3, 10], scientific data on BW remain sparse, with few studies addressing its injury epidemiology explicitly [11, 12].

No published study has yet provided a direct, systematic comparison of injury incidence, severity, and mechanism between OW and BW within the same competitive season. This represents a critical gap in the expanding field of wrestling medicine, particularly as more athletes and coaches engage across formats and as BW gains international recognition through events such as the Association of National Olympic Committees **(**ANOC) World Beach Games and the UWW Beach Wrestling World Series.

This study addresses that gap by comparing injury patterns in OW and BW using standardized epidemiological surveillance methods during the 2025 competitive season. It draws on medical data from the 2025 Senior World Wrestling Championships and the Beach Wrestling World Series, collected according to International Olympic Committee (IOC) and UWW methodologies for injury and illness surveillance in elite sport [8]. The objectives of this research are to quantify and compare injury incidence, characterize anatomical and mechanistic patterns, and evaluate the severity of injuries across the two wrestling formats. Based on differences in competition structure and environment, we hypothesize that BW will demonstrate lower injury severity and a distinct anatomical distribution compared to OW.

By conducting the first comparative analysis of wrestling injury epidemiology across these two formats, this study contributes new evidence to inform prevention strategies, rule modifications, and athlete health protection within UWW’s medical and policy framework.

## Methods

### Study design

This study employed a prospective, observational, epidemiological design to evaluate and compare OW and BW injuries sustained during elite senior international wrestling competitions in 2025: the Senior Olympic-style Wrestling World Championships (WWCh) and the Beach Wrestling World Series (BWWS). Both top-tier events were sanctioned by UWW, which provided standardized medical supervision, centralized injury records, and uniform data collection protocols across competitions.

### Competition structure and wrestling disciplines

Olympic Wrestling consists of three disciplines, Greco-Roman (GR), Freestyle (FS), and Women’s Wrestling (WW), each contested across ten weight categories. Matches are held on a regulated wrestling mat and structured as two three-minute periods, with rules permitting or prohibiting leg attacks depending on the style [10, 13]. In contrast, BW is competed on natural sand under simplified rules that emphasize takedowns, pushouts, and controlled throws, with no prolonged par terre phase. Each bout consists of a single three-minute period, and four weight categories per gender are used at the international level [9, 11].

### Participants

All registered senior athletes competing in either tournament were included in the surveillance cohort. Senior athletes were defined as competitors aged ≥ 18 years, with no upper age limit, although most participants were 18–35 years old.

Each participation was considered as one exposure. The 2025 BWWS consisted of four international stages and a final event, involving a total of 399 wrestlers and 712 bouts. The 2025 WWCh, held in Zagreb, Croatia, featured 776 wrestlers competing in 900 bouts. Only injuries that occurred during active match participation and were evaluated by the official medical team were included. Injuries occurring during training sessions before or after competition were not recorded, and training exposure was not measured.

### Injury definition

An injury was defined as any musculoskeletal or systemic complaint sustained during competition that required medical attention by a licensed medical provider, irrespective of whether the athlete continued to compete. This definition follows the 2020 IOC consensus statement and the associated STROBE extension for sport injury epidemiology [8].

### Injury severity classification

**Fig. 1:**
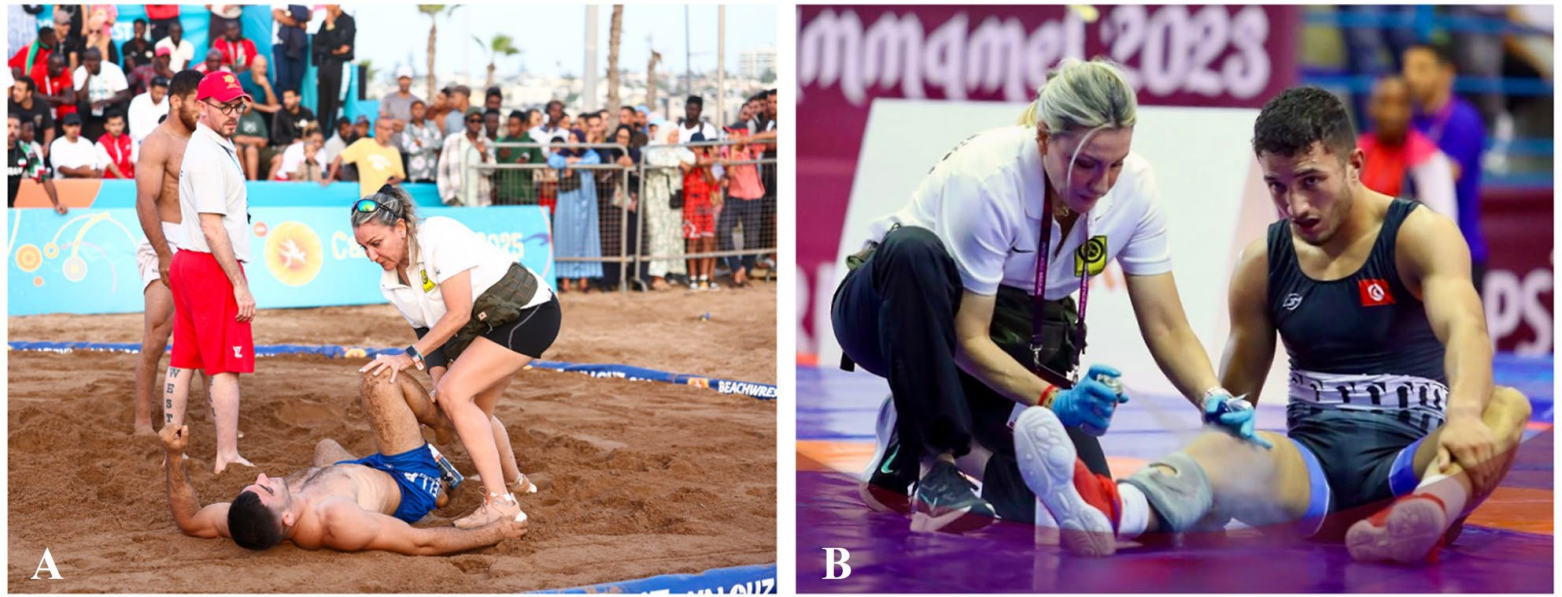
On-site medical care provided by United World Wrestling (UWW) physicians during (**A**) Beach Wrestling and (**B**) Olympic Wrestling competitions

Injury severity was classified using the UWW competition-based operational severity framework rather than exact time-loss–based classifications recommended by the IOC consensus statement [14, 15]. Severity categories were assigned by UWW-appointed event physicians (Fig. 1) at the time of clinical assessment, based on treatment requirements and immediate competition consequences (e.g., continuation or withdrawal from the bout), rather than exact post-event time-loss duration. A mild injury was treated directly on the mat without necessitating withdrawal. Moderate injuries required further medical evaluation or treatment after the bout but permitted continued participation in the later phase of the competition. Severe injuries resulted in match termination and/or referral for hospital care with an anticipated absence of more than one day. Catastrophic injuries were defined as life-threatening or permanently disabling, such as spinal cord injury or cardiopulmonary arrest [3, 14, 15].

### Injury surveillance and data collection

Injury data were recorded on the official United World Wrestling (UWW) Injury Report Form [15] and entered into the UWW Injury Surveillance System [16] within 12 h of the event by licensed tournament physicians. Data collected included wrestler demographics, wrestling discipline, weight category, phase of competition, anatomical injury location, injury type, mechanism of injury, clinical severity, on-site treatment, and referral status. Injury mechanisms were determined by the attending UWW event physician based on direct observation of the injury event when possible, immediate clinical assessment, and athlete report; systematic video analysis was not performed.

Injury diagnoses were recorded based on field-based clinical assessments, without systematic post-event diagnostic confirmation. As a result, diagnostic precision may be limited, particularly for injuries requiring advanced imaging, and injury classification should be interpreted as reflecting acute presentation rather than definitive diagnosis. All recording physicians were trained in the standardized UWW injury reporting protocol prior to event participation to promote inter-observer and inter-event consistency [17, 18].

### Exposure metrics and incidence calculation

Injury incidence was expressed as injuries per 100 athletes, per 100 bouts, and per 1,000 athlete-exposures (AEs), consistent with previously published wrestling injury surveillance methodologies [3, 10]. One athlete-exposure was defined as one athlete participating in one competitive bout, in accordance with the IOC’s general epidemiological recommendations [8]. These metrics allowed comparison across competition formats and with prior epidemiological studies in wrestling.

### Statistical analysis

Data analysis was performed using SPSS Statistics version 29 (IBM Corp., Armonk, NY, USA). Descriptive statistics were used to summarize injury frequency, anatomical distribution, and severity. Chi-square tests (or Fisher’s exact tests, when appropriate) were employed to compare categorical variables, including injury severity and type, between wrestling styles and genders. Independent-samples t-tests were used to compare injury incidence between disciplines and competition phases. A p-value of less than 0.05 was considered statistically significant, and effect sizes were reported using Cramer’s V and Cohen’s d for categorical and continuous data, respectively.

## Results

### Participant and exposure profile

A total of 399 (34%) athletes participated in the 2025 Beach Wrestling World Series (BWWS), competing in 712 (44%) bouts. The 2025 Senior Olympic Wrestling World Championships (WWCh) hosted 776 (66%) wrestlers in 900 (56%) bouts. All injuries occurred during active match participation and were recorded in accordance with UWW surveillance standards.

### Overall injury incidence

Across both formats, 53 competition-related injuries were documented, including 29 injuries in BW and 24 injuries in OW. When expressed per 1,000 AEs, BW demonstrated a higher overall injury incidence than OW (20.4 vs. 13.3 injuries per 1,000 AEs; t [1119] = 3.09, *p* = 0.002; Cohen’s d = 0.26). This pattern was consistent across alternative exposure metrics. BW sustained 29 injuries across 708 bouts (4.1%), compared with 24 injuries across 889 bouts (2.7%) in OW. When expressed per competitor, BW recorded 29 injuries among 402 athletes (7.2%), whereas OW recorded 24 injuries among 774 athletes (3.1%) (Table 1).

**Table 1:** Participant and injury overview for the 2025 competitive season

Discipline	Competitors	Bouts	Injuries	Injuries / wrestler	Injuries / bout	Injuries / 1000 AEs
**Beach wrestling (BW)**	399 (34%)	712 (44%)	29 (54.7%)	7.2%	4.1%	20.4
**Olympic wrestling (OW)**	776 (66%)	900 (56%)	24 (45.3%)	3.1%	2.7%	13.3
**Total**	1175	1621	53	4.5%	3.3%	16.3

Values are presented as n (%) unless otherwise stated. AE = athlete-exposure, defined as one athlete participating in one competitive bout

### Injury severity

Significant differences were observed in injury severity distribution between formats (χ² [2] = 14.73, *p* < 0.001; Cramer’s V = 0.48). In BW, 25 of 29 injuries (86%) were classified as mild and 4 (14%) as moderate, with no severe or catastrophic injuries recorded. In contrast, OW demonstrated a substantially higher proportion of severe injuries: 14 of 24 (58%) were classified as moderate and 4 (17%) as severe, whereas mild injuries accounted for 6 cases (25%) (Table 2). No catastrophic injuries were reported in either format.

**Table 2:** Injury severity distribution by wrestling discipline

Severity	Beach wrestling (*n* = 29)	Olympic wrestling (*n* = 24)
**Mild**	25 (86%)	6 (25%)
**Moderate**	4 (14%)	14 (58%)
**Severe**	0 (0%)	4 (17%)
**Catastrophic**	0 (0%)	0 (0%)

Values are presented as n (%) unless otherwise stated

### Injury distribution by wrestling style (Olympic wrestling)

Within OW, Greco-Roman accounted for 11 of 24 injuries (45.8%), Freestyle for 9 injuries (37.5%), and Women’s Wrestling for 4 injuries (16.7%). Notably, all severe injuries (4 injuries; 100%) occurred in Freestyle and Women’s Wrestling, whereas no severe injuries were recorded in Greco-Roman. A significant difference in the distribution of injury severity across OW styles was observed (χ² [2] = 6.22, *p* = 0.045), with Greco-Roman demonstrating a comparatively milder injury profile (Table 3).

**Table 3:** Injury distribution by wrestling style at the 2025 World championships

Style	Mild	Moderate	Severe	Total
**Greco-roman**	3	8	0	11 (45.8%)
**Freestyle**	3	4	2	9 (37.5%)
**Women’s**	0	2	2	4 (16.7%)
**Total**	6 (25%)	14 (58.3%)	4 (16.7%)	24

Values are presented as n (%) unless otherwise stated

### Anatomical distribution of injuries

In both formats, the head and face were the most commonly injured regions, accounting for 11 of 29 injuries in BW (38%) and 10 of 24 injuries in OW (42%). Injuries to the extremities were also frequent, although their distribution differed between formats.

In BW, elbow injuries (3; 10.3%) and hand/finger injuries (3; 10.3%) were most commonly observed. In contrast, OW uniquely demonstrated ankle/foot injuries (1; 4.2%), which were not observed in BW. Injuries involving the knee, thigh, leg, shoulder, and elbow were present in both formats, with differing distributions across anatomical regions. Detailed anatomical counts and distributions are provided in Table 4.

**Table 4:** Anatomical location of injuries by discipline, in order of frequency

Anatomical region	Beach wrestling *N* (%)		Olympic wrestling *N* (%)	
**Head/face**	11	38%	10	42%
**Knee/thigh/leg**	5	17.2%	6	25%
**Shoulder**	4	13.8%	4	17%
**Torso/ribs/neck**	3	10.3%	3	12.5%
**Elbow/forearm**	3	10.3%	0	0
**Hand/fingers**	3	10.3%	0	0
**Ankle/foot**	0	0%	1	4.2%

### Mechanism of injury

Injury mechanisms differed between formats. In BW, injuries were most associated with slip–fall events and rotational strain during push-out actions, often occurring on the unstable sand surface. In OW, injuries were more frequently attributed to high-force takedowns, leg-attack defences, and upper-body throwing techniques performed on a rigid mat.

## Discussion

### Principal findings and comparative overview

This study presents the first same-season epidemiological comparison of injury patterns in elite senior OW and BW using a unified injury surveillance framework. The findings demonstrate clear format-specific differences. Although BW exhibited a higher overall injury incidence per 1,000 athlete-exposures, all recorded injuries were classified as mild or moderate, with no severe or catastrophic cases observed. In contrast, OW sustained fewer total injuries but a substantially higher proportion of moderate and severe injuries, consistent with prior world-level wrestling injury surveillance reports [2, 3, 10, 19].

These results highlight the importance of interpreting injury incidence alongside injury severity, as incidence alone does not adequately reflect clinical relevance, medical resource utilization, or potential long-term health implications. When considered together, the incidence–severity profile suggests meaningful differences in injury characteristics between the two formats. The observed patterns are consistent with known structural distinctions between OW and BW, including differences in bout structure, rule constraints, and competition environment, although these factors were not directly measured in the present study.

Collectively, the findings support the hypothesis that injury risk in combat sports is influenced not only by exposure volume but also by competition-format-specific characteristics. Within the limits of the available data, this comparative overview underscores the value of standardized surveillance approaches for identifying format-dependent injury patterns and informing targeted injury-prevention strategies.

### Comparability with previous wrestling injury epidemiology

Injury is a significant barrier to sustained participation in combat sports and to athletic development. Although wrestling injuries have been widely investigated, substantial heterogeneity persists across studies in injury definitions, surveillance methods, exposure metrics, and athlete populations, complicating direct inter-study comparisons [1–3].

The injury incidence observed in OW in the present study (13.3 injuries per 1,000 AEs) aligns with the lower end of rates previously reported in surveillance of elite Olympic-style wrestling at World Championships and Olympic Games. When OW and BW were combined, the overall injury incidence (16.3 injuries per 1,000 AEs) remained within the lower boundary of published ranges for international wrestling competitions (approximately 16.3–69.5 injuries per 1,000 AEs), supporting the external validity of the observed incidence estimates [3].

Despite comparable incidence rates, injury severity distributions differed substantially across wrestling formats and competition levels. In BW, mild injuries accounted for 86% of all recorded cases, a proportion comparable to that reported during the Olympic Games (67.3%) but markedly higher than that observed during OW World Championships (25%). Moderate injuries comprised 14% of BW injuries, compared with 58.3% at the OW World Championships and 18.3% at the Olympic Games. Notably, no severe injuries were observed in BW, whereas severe injuries accounted for 17% of injuries at the World Championships and 14.3% at the Olympic Games [3]. These findings reinforce prior observations that injury incidence alone does not adequately reflect clinical burden and that competition format plays a critical role in shaping injury severity profiles.

Differences in injury severity may be partly attributable to structural and competitive characteristics of the events. The OW World Championships involve substantially larger athlete cohorts and competition volumes than the Olympic Games (776 athletes and approximately 900 bouts in the present study versus 287 athletes and 322 bouts in Tokyo, and 346 athletes and 410 bouts in Rio). In addition, World Championship formats may require athletes to compete in up to five or six bouts on the opening day of competition, compared with a maximum of three bouts per day at the Olympic Games. This higher bout density and cumulative load may contribute to increased fatigue and a greater likelihood of moderate-to-severe injuries [2, 3].

The distribution of injuries by wrestling style at the World Championships closely mirrored patterns reported at the Olympic Games. Greco-Roman wrestling accounted for 45.8% of injuries in the present study, compared with 41.6% in Tokyo and Rio; Freestyle wrestling accounted for 37.5% versus 36.7%; and Women’s Wrestling for 16.6% versus 22.5% [3]. Across both OW and BW, anatomical injury patterns were broadly similar across wrestling styles and competition formats. The head and face were the most frequently injured regions (BW 38%; OW World Championships 42%; Olympic Games 73.5%), followed by the lower extremities, including the knee, thigh, leg, ankle, and foot (BW 17.2%; OW 29.2%; Olympic Games 14.3%), and the upper extremities (BW 34.6%; OW 29.5%; Olympic Games 10.2%). Injuries involving the trunk, ribs, neck, or spine were consistently least frequent across all competition formats (BW 10.3%; OW 12.5%; Olympic Games 2%) [3]. These comparisons provide essential context for interpreting the format-specific differences observed in the present study and inform the mechanistic considerations discussed below.

### Mechanistic and structural contributors to injury differences

The distinct injury profiles observed between BW and OW may be influenced by several structural and contextual differences between formats, including bout structure, competition scheduling, technical demands, weight-management practices, and competition surface characteristics. Although these factors were not directly measured in the present study, they provide a plausible framework for interpreting the observed incidence–severity patterns.

First, BW bouts consist of a single three-minute period, whereas OW bouts comprise two three-minute periods. Shorter bout duration and more condensed tournament schedules in BW may reduce cumulative exposure and neuromuscular fatigue, both of which have been associated with increased injury risk in high-intensity combat sports [20]. In addition, BW competitions are typically completed within one to two days, compared with the four- to five-day duration of OW events, potentially limiting cumulative load and recovery-related vulnerability [3].

Second, the technical and tactical demands differ between formats. BW rules emphasize positional control, balance disruption, and push-out actions, whereas OW involves higher point accumulation through forceful leg attacks and high-amplitude throws. These OW-specific techniques impose substantial joint loading and rotational stress, particularly at the knee and shoulder, and align with injury patterns reported in prior OW surveillance studies [5, 14]. Such differences may contribute to the higher proportion of moderate and severe injuries observed in OW, although causal inference cannot be established from the present data.

Third, BW athletes may compete at body weights closer to their habitual mass, given fewer weight categories and reduced incentives for rapid weight loss. Reduced reliance on aggressive weight-cutting practices could mitigate dehydration-related neuromuscular impairment and physiological vulnerability, which have been associated with increased injury risk in OW populations [21]. However, individual weight-management behaviours were not assessed in this study.

Finally, the competition surface represents a key contextual difference. OW is performed on a rigid mat, whereas BW takes place on sand, which deforms on impact and dissipates mechanical energy. Experimental biomechanical studies have shown that sand surfaces reduce peak impact forces, vertical loading rates, and joint shock compared with rigid flooring [22, 23]. Consistent with epidemiological findings from other sand-based sports, where severe traumatic injuries are less frequent despite similar exposure to falls, this energy-absorbing property may contribute to the absence of severe injuries observed in BW [24]. Nevertheless, surface biomechanics were not directly evaluated and should be interpreted as a contextual factor rather than a measured determinant.

### Comparisons with other dual-format Olympic and combat sports

The injury patterns observed between OW and BW are consistent with trends reported in other Olympic and Olympic-style sports that feature both traditional indoor and sand-based competition formats. Across these disciplines, overall injury incidence is often comparable between formats; however, sand-based competitions consistently demonstrate a lower proportion of high-energy traumatic injuries. This pattern has been attributed to differences in surface compliance, match structure, and technical demands rather than exposure volume alone.

In volleyball, beach competition has been associated with similar or slightly higher rates of minor injuries related to surface instability, while severe injuries, such as ankle sprains, knee ligament ruptures, and concussions, occur more frequently during indoor play on rigid courts [25, 26]. Similar observations have been reported in handball, where indoor competition is associated with higher rates of anterior cruciate ligament injury, shoulder trauma, and collision-related injuries, whereas beach handball is characterized by reduced joint loading due to shorter match duration, modified rules, and the energy-absorbing properties of sand [24, 27].

Comparable format-dependent injury patterns are increasingly recognized in combat and grappling sports. Sand-based variants of sambo and emerging adaptations of judo employ modified rule sets, shorter bout durations, and compliant surfaces that limit high-risk groundwork and attenuate fall-related impact forces. These design features are intended to reduce the likelihood of severe joint and head trauma while preserving competitive intensity. Within this broader dual-format paradigm, wrestling demonstrates a similar pattern, whereby differences in competition surface, bout structure, and permitted techniques appear to influence injury severity more strongly than overall injury frequency.

Collectively, evidence from multiple sports supports a cross-disciplinary principle that energy-absorbing competition environments and format-specific rule modifications are associated with a lower risk of clinically severe injury, even when overall exposure remains similar. The present findings situate BW within this framework and reinforce the importance of competition design as a modifiable factor in injury prevention across combat sports.

### Implications for athlete safety, sport development, and injury prevention

The contrasting injury profiles observed between OW and BW have important implications for athlete safety, sport development, and governance. The absence of severe injuries in BW, despite a higher overall injury incidence, suggests a lower injury-severity burden among competition-related injuries captured in this study. This pattern indicates that BW may represent a comparatively safer competitive environment with respect to injury severity while preserving key biomechanical and tactical elements of wrestling. As such, BW may be particularly well suited for youth development pathways, recreational participation, and athletes with increased medical vulnerability related to prior injury or weight-management stress, although these applications were not directly evaluated.

At the same time, BW is not injury-free. The predominance of mild injuries, often associated with surface instability and rotational movements, underscores the need for targeted preventive strategies. These may include surface-specific warm-up routines, proprioceptive and balance training, and competition-appropriate medical preparedness tailored to the demands of sand-based competition.

In contrast, the higher proportion of moderate and severe injuries observed in OW highlights the need for focused injury-prevention strategies addressing high-force mechanisms, particularly knee and shoulder trauma associated with takedowns, defensive actions, and rotational throws. Preventive efforts should emphasize neuromuscular conditioning, careful monitoring during periods of rapid weight loss, and continued evaluation of competition structures and mat technologies that may reduce cumulative biomechanical load over multi-day tournaments.

From a governance perspective, these findings support a differentiated approach to injury prevention across wrestling formats: targeted risk-reduction strategies for OW and continued injury surveillance and risk-containment measures for BW. More broadly, BW illustrates how environmental and structural modifications to competition design may reduce injury severity without compromising competitive integrity. This principle may be increasingly relevant for the evolution of injury-prevention strategies in combat sports, provided that such approaches are supported by ongoing, high-quality surveillance data.

### Strengths and limitations

A significant strength of this study is the use of a standardized, physician-led injury surveillance system applied uniformly across both competitions. All injuries were recorded by trained UWW event physicians using identical reporting protocols, structured injury report forms, and centralized digital data entry, thereby minimizing inter-observer variability, diagnostic ambiguity, and reporting bias. Near-real-time physician reporting, mandated adherence to a common case definition, and identical surveillance infrastructure across formats strengthen internal validity and support attribution of observed differences to true sport-specific factors rather than measurement artifacts. In addition, both competitions occurred within the same competitive season, reducing potential confounding related to seasonal variation in athlete conditioning and weight-management practices. The use of IOC-aligned injury definitions and exposure metrics further facilitates comparison with Olympic- and World-level surveillance datasets.

Several limitations should be considered. Injury severity was classified using the UWW competition-based medical severity framework rather than exact time-loss–based classifications recommended by the IOC consensus statement. While this approach reflects real-time medical decision-making during elite wrestling competitions, severity categorization may be influenced by immediate clinical management decisions and may bias classification toward higher severity categories compared with systems relying on post-event time-loss data. This limits direct comparability with studies applying strict time-loss–based definitions and precludes calculation of injury burden.

Injury surveillance was restricted to competition-related injuries requiring medical attention and did not include training-related injuries or training exposure, which were not systematically recorded. Consequently, the findings should be interpreted as reflecting competition-related injury patterns rather than overall injury risk or total injury burden in wrestling, limiting generalizability to the broader training–competition continuum. Injury incidence could not be expressed per 1,000 player-hours because individual exposure time and bout duration were not consistently documented across all competitions; therefore, incidence was calculated using athlete-exposures, which may further limit comparability with studies employing time-based exposure metrics. The absence of a time-loss requirement may have increased the proportion of recorded mild injuries; however, this definition was applied consistently across formats.

In addition, individual-level risk factors such as age, prior injury history, training load, and competitive experience were not controlled for, and proposed mechanistic explanations are supported by existing biomechanical literature but were not directly measured. This analysis reflects a single competitive season and a relatively small number of recorded injuries, which limited statistical power, particularly for stratified and rate-based analyses, and increased the risk of imprecise estimates and wide confidence intervals. Accordingly, the findings should be interpreted cautiously and viewed as exploratory. To mitigate overinterpretation, results are presented primarily using absolute counts, incidence rates, and effect-size measures (Cramer’s V and Cohen’s d), which convey the magnitude and practical relevance of observed differences independent of statistical significance.

Sex-specific analyses were not performed because Beach Wrestling was evaluated as a unified competition format, and the limited number of injuries recorded within a single competitive season precluded reliable stratified comparisons by sex. More broadly, the small number of injuries reduced statistical power and the precision of effect estimates, particularly for subgroup analyses, and any confidence intervals derived from such analyses would be expected to be wide and should be interpreted with caution.

Larger, multi-season injury surveillance datasets incorporating systematic time-loss follow-up, training exposure, and time-based exposure metrics will be required to support more robust inference, precise confidence interval estimation, and calculation of injury burden.

## Conclusions

This same-season comparative injury surveillance study demonstrates distinct injury patterns between Olympic Wrestling and Beach Wrestling at the elite senior level. Beach Wrestling exhibited a higher overall injury incidence per athlete-exposure; however, all recorded injuries were classified as mild or moderate, with no severe or catastrophic injuries observed. In contrast, Olympic Wrestling sustained a lower overall incidence but a substantially higher proportion of moderate and severe injuries, indicating a greater injury-severity burden.

These findings underscore the importance of evaluating injury severity alongside incidence when comparing injury risk across wrestling formats. The observed divergence in injury profiles highlights the need for format-specific injury surveillance and targeted prevention strategies tailored to the distinct demands of each discipline. Continued standardized injury monitoring across multiple seasons will be essential to confirm these patterns, increase the certainty of evidence, and inform evidence-based policy and injury-prevention initiatives in international wrestling.

## Data Availability

The datasets used and analysed during the current study are available from the corresponding author or from the contact person of the funding organisation on reasonable request.
